# Supplementary material for: Synthesis, Characterization, and Non-Covalent Interactions of Palladium(II)-Amino Acid Complexes
Source: Molecules. 2021 Jul 17;26(14):4331. doi: 10.3390/molecules26144331 (PMC8303679; doi:10.3390/molecules26144331)
Supplement: Supplementary file 1 [file molecules-26-04331-s001.zip › molecules-1268646-supplementary -layout.pdf]

molecules

Supplementary Material

Synthesis, Characterization, and Non-Covalent Interactions of Palladium(II)-Amino Acid Complexes

David B. Hobart, Jr. [1,2], Michael A. G. Berg [1], Hannah M. Rogers [1] and Joseph S. Merola [1,*]

[1] Department of Chemistry, Virginia Polytechnic Institute & State University, Blacksburg, VA 24061 USA
[2] Current Address: Air Liquide Advanced Materials, 197 Meister Ave., Branchburg, NJ 08876 USA
* Correspondence: jmerola@vt.edu

Contents:	Page
A. Experimental data and all tables related to the crystal structure of complex (1)	2
B. Experimental data and all tables related to the crystal structure of complex (2)	5
C. Experimental data and all tables related to the crystal structure of complex (4)	9
D. Experimental data and all tables related to the crystal structure of complex (15)	13
E. Experimental data and all tables related to the crystal structure of complex (16)	16
F. Experimental data and all tables related to the crystal structure of complex (17)	21

A. Complex (1) *trans*-bis-(alaninato)palladium(II)

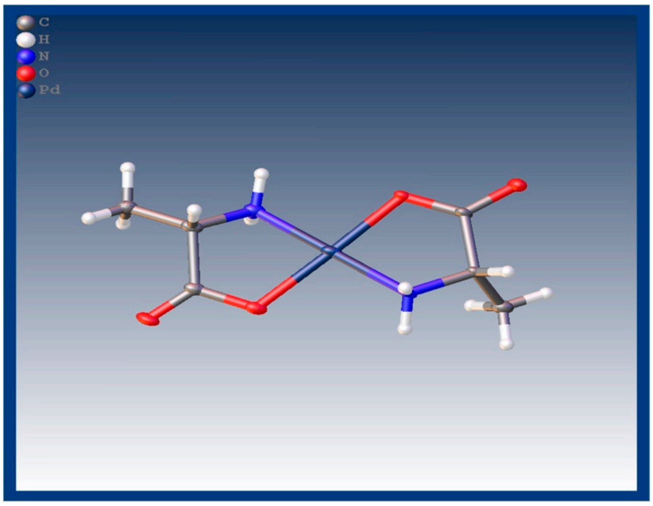

Table S1. Crystal data and structure refinement for Pd_alanine.

Identification code	Pd_alanine
Empirical formula	$C_6H_{12}N_2O_4Pd$
Formula weight	282.58
Temperature/K	99.8(3)
Crystal system	triclinic
Space group	P1
a/Å	4.6540(5)
b/Å	5.1589(6)
c/Å	9.4786(9)
α/°	79.048(8)
β/°	85.410(8)
γ/°	75.629(10)
Volume/Å^3	216.31(4)
Z	1
ρ_{calc}g/cm^3	2.169
μ/mm^{-1}	2.129
F(000)	140.0
Crystal size/mm^3	0.3 × 0.163 × 0.087
Radiation	Mo Kα (λ = 0.71073)
2Θ range for data collection/°	8.286 to 64.628
Index ranges	$-7 \le h \le 6$, $-7 \le k \le 7$, $-13 \le l \le 13$
Reflections collected	4326
Independent reflections	2801 [R_{int} = 0.0457, R_{sigma} = 0.0975]
Data/restraints/parameters	2801/3/84
Goodness-of-fit on F^2	0.988
Final R indexes [I>=2σ (I)]	R_1 = 0.0506, wR_2 = 0.0953
Final R indexes [all data]	R_1 = 0.0587, wR_2 = 0.1017
Largest diff. peak/hole / e Å^{-3}	1.82/-2.62
Flack parameter	-0.07(10)

Table S2. Fractional Atomic Coordinates (×10[4]) and Equivalent Isotropic Displacement Parameters (Å[2]×10[3]) for Pd_alanine. U_{eq} is defined as 1/3 of of the trace of the orthogonalised U_{IJ} tensor.

Atom	*x*	*y*	*z*	U(eq)
Pd1	4817(8)	4777(7)	4742(5)	15.07(16)
O1	8590(30)	2620(30)	3875(17)	15.6(19)
O2	11950(40)	2960(40)	2200(20)	19(3)
O3	1230(30)	6980(30)	5547(16)	12.9(17)
O4	-2090(40)	6840(40)	7430(20)	18(3)
N1	6090(40)	7850(40)	3380(20)	15.6(19)
N2	3480(40)	1640(40)	6060(20)	12.9(17)
C1	9610(40)	3920(40)	2710(20)	15.6(19)
C2	7550(20)	6650(20)	2119(14)	15.6(19)
C3	9120(20)	8500(20)	1089(12)	18.4(18)
C4	90(40)	5750(40)	6680(20)	12.9(17)
C5	1500(20)	2720(20)	7235(13)	12.9(17)
C6	3190(20)	2440(20)	8558(12)	19.8(18)

Table S3. Anisotropic Displacement Parameters (Å[2]×10[3]) for Pd_alanine. The Anisotropic displacement factor exponent takes the form: $-2\pi^2[h^2a^{*2}U_{11}+2hka^*b^*U_{12}+\ldots]$.

Atom	U_{11}	U_{22}	U_{33}	U_{23}	U_{13}	U_{12}
Pd1	11.2(2)	10.4(2)	22.1(3)	-2.71(18)	-1.45(19)	0.41(16)
O1	11(3)	12(4)	23(4)	-3(3)	-2(3)	-1(3)
O2	11(5)	20(6)	24(7)	-5(5)	-3(4)	4(4)
O3	10(3)	9(3)	18(4)	-2(3)	1(2)	1(2)
O4	17(6)	16(5)	16(6)	-1(4)	1(4)	4(4)
N1	11(3)	12(4)	23(4)	-3(3)	-2(3)	-1(3)
N2	10(3)	9(3)	18(4)	-2(3)	1(2)	1(2)
C1	11(3)	12(4)	23(4)	-3(3)	-2(3)	-1(3)
C2	11(3)	12(4)	23(4)	-3(3)	-2(3)	-1(3)
C3	17(4)	14(4)	21(5)	-1(3)	0(4)	0(3)
C4	10(3)	9(3)	18(4)	-2(3)	1(2)	1(2)
C5	10(3)	9(3)	18(4)	-2(3)	1(2)	1(2)
C6	16(4)	16(4)	23(5)	-1(4)	-2(4)	2(3)

Table S4. Bond Lengths for Pd_alanine.

Atom	Atom	Length/Å	Atom	Atom	Length/Å
Pd1	O1	2.036(15)	O4	C4	1.27(3)
Pd1	O3	1.962(14)	N1	C2	1.49(2)
Pd1	N1	2.02(2)	N2	C5	1.49(2)
Pd1	N2	2.04(2)	C1	C2	1.53(2)
O1	C1	1.30(2)	C2	C3	1.509(15)
O2	C1	1.18(3)	C4	C5	1.54(2)
O3	C4	1.29(2)	C5	C6	1.498(14)

Table S5. Bond Angles for Pd_alanine.

Atom	Atom	Atom	Angle/˚	Atom	Atom	Atom	Angle/˚
O1	Pd1	N2	99.0(7)	O2	C1	O1	120(2)
O3	Pd1	O1	177.9(8)	O2	C1	C2	125.9(19)
O3	Pd1	N1	97.3(7)	N1	C2	C1	106.9(15)
O3	Pd1	N2	83.1(8)	N1	C2	C3	113.6(11)

Atom	Atom	Atom	Angle/°	Atom	Atom	Atom	Angle/°
N1	Pd1	O1	80.6(8)	C3	C2	C1	112.8(11)
N1	Pd1	N2	178.1(10)	O3	C4	C5	119.1(16)
C1	O1	Pd1	114.6(14)	O4	C4	O3	125.2(18)
C4	O3	Pd1	114.8(13)	O4	C4	C5	115.7(16)
C2	N1	Pd1	105.6(12)	N2	C5	C4	107.5(14)
C5	N2	Pd1	109.0(12)	N2	C5	C6	111.4(10)
O1	C1	C2	114.0(17)	C6	C5	C4	110.0(11)

Table S6. Torsion Angles for Pd_alanine.

A	B	C	D	Angle/°	A	B	C	D	Angle/°
Pd1	O1	C1	O2	172.5(18)	O1	C1	C2	N1	35.8(18)
Pd1	O1	C1	C2	-7.8(19)	O1	C1	C2	C3	161.3(13)
Pd1	O3	C4	O4	174.5(19)	O2	C1	C2	N1	-145(2)
Pd1	O3	C4	C5	-3(2)	O2	C1	C2	C3	-19(3)
Pd1	N1	C2	C1	-45.1(13)	O3	C4	C5	N2	-16(2)
Pd1	N1	C2	C3	-170.1(8)	O3	C4	C5	C6	105.7(17)
Pd1	N2	C5	C4	25.4(14)	O4	C4	C5	N2	166.4(18)
Pd1	N2	C5	C6	-95.3(12)	O4	C4	C5	C6	-72(2)

Table S7. Hydrogen Atom Coordinates (Å×10^4) and Isotropic Displacement Parameters (Å^2×10^3) for Pd_alanine.

Atom	*x*	*y*	*z*	U(eq)
H1A	4525.21	9201.27	3115.37	19
H1B	7353.54	8481.74	3799.42	19
H2A	2504.5	857.52	5556.61	15
H2B	5049.1	399.98	6426.45	15
H2	6003.71	6289.37	1592.39	19
H3A	10677.55	8860.58	1574.06	28
H3B	7733.7	10178.51	749.27	28
H3C	9962.14	7635.36	288.37	28
H5	-68.24	1728.35	7467.85	15
H6A	4157.16	558.46	8864.9	30
H6B	1850.7	3074.35	9308.89	30
H6C	4657.78	3490.52	8347.49	30

Experimental

Single crystals of $C_6H_{12}N_2O_4Pd$ [Pd_alanine] were **grown by allowing an acetone/water solution to evaporate slowly**. A suitable crystal was selected and **mounted on a fiber** on a **Xcalibur, Sapphire3, Gemini ultra** diffractometer. The crystal was kept at 99.8(3) K during data collection. Using Olex2 [1], the structure was solved with the XS [2] structure solution program using Direct Methods and refined with the XL [3] refinement package using Least Squares minimisation.

1. Dolomanov, O.V., Bourhis, L.J., Gildea, R.J, Howard, J.A.K. & Puschmann, H. (2009), J. Appl. Cryst. 42, 339-341.
2. Sheldrick, G.M. (2008). Acta Cryst. A64, 112-122.
3. Sheldrick, G.M. (2008). Acta Cryst. A64, 112-122.

Crystal structure determination of [Pd_alanine]

Crystal Data for $C_6H_{12}N_2O_4Pd$ (M =282.58 g/mol): triclinic, space group P1 (no. 1), a = 4.6540(5) Å, b = 5.1589(6) Å, c = 9.4786(9) Å, α = 79.048(8)°, β = 85.410(8)°, γ = 75.629(10)°, V = 216.31(4) Å^3, Z = 1, T = 99.8(3) K, μ(Mo Kα) = 2.129 mm^{-1}, $Dcalc$ = 2.169 g/cm^3, 4326 reflections measured (8.286° ≤ 2Θ ≤ 64.628°), 2801 unique (R_{int} = 0.0457, R_{sigma} = 0.0975) which were used in all calculations. The final R_1 was 0.0506 (I > 2σ(I)) and wR_2 was 0.1017 (all data).

Refinement model description

Number of restraints - 3, number of constraints - unknown.

Details:

```
1. Fixed Uiso
 At 1.2 times of:
  All C(H) groups, All N(H,H) groups
 At 1.5 times of:
  All C(H,H,H) groups
2. Uiso/Uaniso restraints and constraints
Uanis(N2) = Uanis(C5) = Uanis(C4) = Uanis(O3)
Uanis(O1) = Uanis(C1) = Uanis(C2) = Uanis(N1)
3.a Ternary CH refined with riding coordinates:
 C2(H2), C5(H5)
3.b Secondary CH2 refined with riding coordinates:
 N1(H1A,H1B), N2(H2A,H2B)
3.c Idealised Me refined as rotating group:
 C3(H3A,H3B,H3C), C6(H6A,H6B,H6C)
```

B. Complex (2) *cis*-bis-(valinato)palladium(II)

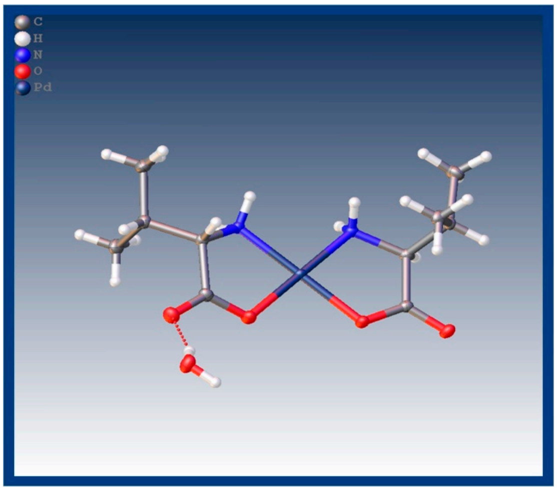

Table S1. Crystal data and structure refinement for valinepd.

Identification code	valinepd
Empirical formula	$C_{10}H_{22}N_2O_5Pd$
Formula weight	356.69
Temperature/K	100.05(10)
Crystal system	orthorhombic
Space group	$P2_12_12_1$
a/Å	9.57160(10)
b/Å	9.61940(10)
c/Å	15.22950(10)
α/°	90
β/°	90
γ/°	90
Volume/Å^3	1402.23(2)
Z	4
ρ_{calc}g/cm^3	1.690
μ/mm^{-1}	1.338
F(000)	728.0
Crystal size/mm^3	0.263 × 0.197 × 0.144
Radiation	Mo Kα (λ = 0.71073)
2Θ range for data collection/°	8.474 to 65.308
Index ranges	$-14 \leq h \leq 14$, $-14 \leq k \leq 14$, $-22 \leq l \leq 22$
Reflections collected	27728
Independent reflections	4869 [R_{int} = 0.0315, R_{sigma} = 0.0178]
Data/restraints/parameters	4869/0/170

Goodness-of-fit on F^2	1.121
Final R indexes [I>=2σ (I)]	R_1 = 0.0168, wR_2 = 0.0395
Final R indexes [all data]	R_1 = 0.0172, wR_2 = 0.0397
Largest diff. peak/hole / e Å^{-3}	0.63/-0.62
Flack parameter	-0.024(8)

Table S2. Fractional Atomic Coordinates (×10^4) and Equivalent Isotropic Displacement Parameters (Å^2×10^3) for valinepd. U_{eq} is defined as 1/3 of of the trace of the orthogonalised U_{IJ} tensor.

Atom	*x*	*y*	*z*	U(eq)
Pd1	5391.9(2)	3952.7(2)	6631.6(2)	11.54(4)
O1	4672.2(16)	2814.8(14)	7647.9(9)	15.6(2)
O2	3022.6(16)	2743.4(15)	8675.3(10)	17.4(3)
O3	6958.2(16)	2663.1(16)	6313.2(10)	18.5(3)
O4	8439.7(16)	2158.6(16)	5233.9(11)	19.8(3)
N1	3827.9(17)	5250.1(16)	6957.2(10)	12.3(3)
N2	6112.8(17)	5053.1(16)	5608.7(10)	12.5(3)
C1	3580.9(19)	3313.4(19)	8039.0(12)	13.0(3)
C2	2962(2)	4673.6(19)	7689.2(12)	12.9(3)
C3	1433.7(19)	4437(2)	7413.8(13)	14.5(3)
C4	1318(2)	3354(2)	6684.6(14)	17.9(3)
C5	740(2)	5811(2)	7155.4(15)	22.7(4)
C6	7506(2)	2891.3(19)	5550.3(13)	14.9(3)
C7	6887.5(18)	4093.0(19)	5014.3(12)	13.2(3)
C8	7945(2)	4830(2)	4414.8(13)	16.0(3)
C9	7208(2)	5892(3)	3832.1(14)	23.8(4)
C10	9135(2)	5487(2)	4932.7(15)	20.6(4)
O1W	8330.0(17)	-659.5(15)	4998.8(11)	20.1(3)

Table S3. Anisotropic Displacement Parameters (Å^2×10^3) for valinepd. The Anisotropic displacement factor exponent takes the form: $-2\pi^2[h^2a^{*2}U_{11}+2hka^*b^*U_{12}+\ldots]$.

Atom	U_{11}	U_{22}	U_{33}	U_{23}	U_{13}	U_{12}
Pd1	12.15(5)	9.94(5)	12.53(5)	1.77(4)	0.19(5)	0.84(5)
O1	16.4(6)	13.8(6)	16.5(6)	4.5(4)	3.5(5)	3.7(5)
O2	21.9(7)	15.3(6)	15.0(6)	3.5(5)	4.1(5)	2.5(5)
O3	19.1(7)	16.1(6)	20.4(7)	4.6(5)	4.7(5)	6.1(5)
O4	19.0(7)	15.9(6)	24.6(7)	-1.1(6)	3.5(6)	3.8(5)
N1	12.7(6)	9.8(6)	14.3(6)	1.4(5)	-0.7(5)	0.1(5)
N2	13.4(6)	10.0(6)	14.2(6)	1.4(5)	1.2(6)	-0.5(5)
C1	14.8(8)	11.3(7)	12.9(7)	0.3(6)	-1.0(6)	0.1(6)
C2	15.5(8)	11.5(7)	11.7(7)	-0.5(6)	-0.7(6)	0.9(6)
C3	12.3(8)	16.5(8)	14.8(8)	0.8(6)	1.5(6)	0.8(6)
C4	17.2(8)	16.8(8)	19.7(8)	0.5(7)	-1.5(8)	-4.3(6)
C5	20.1(9)	22.7(10)	25.3(9)	-0.8(8)	-1.2(7)	8.2(7)
C6	14.4(8)	11.1(8)	19.2(9)	-0.5(6)	0.1(7)	1.0(6)
C7	13.7(7)	11.9(7)	14.1(7)	0.1(6)	0.7(6)	0.4(6)
C8	15.1(8)	17.2(8)	15.7(8)	0.7(7)	1.8(7)	0.1(7)
C9	22.0(9)	29.1(11)	20.2(9)	9.6(9)	0.4(7)	0.6(9)
C10	16.7(8)	21.7(9)	23.5(10)	3.1(8)	-0.3(7)	-4.2(7)
O1W	25.1(7)	15.5(6)	19.6(7)	0.5(5)	8.1(6)	-0.5(5)

Table S4. Bond Lengths for valinepd.

Atom	Atom	Length/Å	Atom	Atom	Length/Å
Pd1	O1	2.0170(13)	N2	C7	1.491(2)
Pd1	O3	2.0054(15)	C1	C2	1.532(3)
Pd1	N1	2.0110(16)	C2	C3	1.539(3)
Pd1	N2	2.0058(15)	C3	C4	1.527(3)
O1	C1	1.295(2)	C3	C5	1.531(3)
O2	C1	1.235(2)	C6	C7	1.534(3)
O3	C6	1.293(2)	C7	C8	1.537(3)
O4	C6	1.236(2)	C8	C9	1.526(3)
N1	C2	1.495(2)	C8	C10	1.523(3)

Table S5. Bond Angles for valinepd.

Atom	Atom	Atom	Angle/°	Atom	Atom	Atom	Angle/°
O3	Pd1	O1	96.03(6)	N1	C2	C3	112.24(15)
O3	Pd1	N1	179.65(7)	C1	C2	C3	109.63(15)
O3	Pd1	N2	83.19(6)	C4	C3	C2	111.59(15)
N1	Pd1	O1	83.88(6)	C4	C3	C5	111.78(17)
N2	Pd1	O1	178.98(6)	C5	C3	C2	110.78(16)
N2	Pd1	N1	96.90(6)	O3	C6	C7	116.72(16)
C1	O1	Pd1	115.31(12)	O4	C6	O3	123.12(18)
C6	O3	Pd1	114.53(12)	O4	C6	C7	120.08(18)
C2	N1	Pd1	111.47(11)	N2	C7	C6	109.61(15)
C7	N2	Pd1	108.41(11)	N2	C7	C8	113.75(15)
O1	C1	C2	117.93(16)	C6	C7	C8	114.19(15)
O2	C1	O1	123.07(17)	C9	C8	C7	110.48(16)
O2	C1	C2	119.00(17)	C10	C8	C7	112.11(16)
N1	C2	C1	111.21(15)	C10	C8	C9	111.66(18)

Table S6. Hydrogen Bonds for valinepd.

D	H	A	d(D-H)/Å	d(H-A)/Å	d(D-A)/Å	D-H-A/°
N2	H2A	O1W[1]	0.89	2.06	2.879(2)	153.6
O1W	H1WA	O4	0.85	1.92	2.736(2)	162.1
O1W	H1WB	O2[2]	0.85	2.00	2.848(2)	175.7

[1]-1/2+X,1/2-Y,1-Z; [2]1-X,-1/2+Y,3/2-Z.

Table S7. Torsion Angles for valinepd.

A	B	C	D	Angle/°	A	B	C	D	Angle/°
Pd1	O1	C1	O2	-179.49(15)	O3	C6	C7	N2	19.6(2)
Pd1	O1	C1	C2	0.6(2)	O3	C6	C7	C8	148.52(18)
Pd1	O3	C6	O4	-176.79(15)	O4	C6	C7	N2	-163.47(17)
Pd1	O3	C6	C7	0.1(2)	O4	C6	C7	C8	-34.5(2)
Pd1	N1	C2	C1	4.79(18)	N1	C2	C3	C4	63.2(2)
Pd1	N1	C2	C3	-118.44(14)	N1	C2	C3	C5	-62.0(2)
Pd1	N2	C7	C6	-28.66(16)	N2	C7	C8	C9	-59.7(2)
Pd1	N2	C7	C8	-157.86(12)	N2	C7	C8	C10	65.5(2)
O1	C1	C2	N1	-3.6(2)	C1	C2	C3	C4	-60.9(2)
O1	C1	C2	C3	121.08(18)	C1	C2	C3	C5	173.85(16)
O2	C1	C2	N1	176.44(17)	C6	C7	C8	C9	173.44(17)
O2	C1	C2	C3	-58.9(2)	C6	C7	C8	C10	-61.3(2)

Table S8. Hydrogen Atom Coordinates (Å×10[4]) and Isotropic Displacement Parameters (Å[2]×10[3]) for valinepd.

Atom	*x*	*y*	*z*	U(eq)
H1A	4183.25	6065.38	7119.87	15

Atom	*x*	*y*	*z*	U(eq)
H1B	3287.73	5392.7	6490.18	15
H2A	5404.47	5443.73	5321.63	15
H2B	6678.54	5724.3	5796.77	15
H2	2967.7	5351.52	8169.51	15
H3	933.23	4074.04	7926.21	17
H4A	1798.31	3681.09	6171.41	27
H4B	351.4	3201.22	6546.61	27
H4C	1731.46	2497.13	6877.52	27
H5A	908.73	6491.97	7603.92	34
H5B	-247.87	5672.13	7091.04	34
H5C	1124.16	6130.89	6609.31	34
H7	6186.85	3678.32	4625.06	16
H8	8354.09	4123.73	4028.71	19
H9A	6441.28	5453.18	3535.06	36
H9B	7855.92	6249.07	3406.98	36
H9C	6863.9	6641.23	4187.7	36
H10A	8764.76	6172.39	5327.35	31
H10B	9778.45	5921.36	4534.53	31
H10C	9611.86	4781.36	5262.64	31
H1WA	8252.12	179.06	5167.55	30
H1WB	7964.99	-1133.94	5410.44	30

Experimental

Single crystals of $C_{10}H_{22}N_2O_5Pd$ **[valinepd]** were **grown by allowing an acetone/water solution to evaporate slowly**. A suitable crystal was selected and mounted on fiber on a **Xcalibur, Sapphire3, Gemini ultra** diffractometer. The crystal was kept at 100.05(10) K during data collection. Using Olex2 [1], the structure was solved with the XS [2] structure solution program using Direct Methods and refined with the SHELXL [3] refinement package using Least Squares minimisation.

1. Dolomanov, O.V., Bourhis, L.J., Gildea, R.J, Howard, J.A.K. & Puschmann, H. (2009), J. Appl. Cryst. 42, 339-341.
2. Sheldrick, G.M. (2008). Acta Cryst. A64, 112-122.
3. Sheldrick, G.M. (2015). Acta Cryst. C71, 3-8.

Crystal structure determination of [valinepd]

Crystal Data for $C_{10}H_{22}N_2O_5Pd$ (M =356.69 g/mol): orthorhombic, space group $P2_12_12_1$ (no. 19), a = 9.57160(10) Å, b = 9.61940(10) Å, c = 15.22950(10) Å, V = 1402.23(2) Å^3, Z = 4, T = 100.05(10) K, μ(Mo Kα) = 1.338 mm^{-1}, $Dcalc$ = 1.690 g/cm^3, 27728 reflections measured (8.474° ≤ 2Θ ≤ 65.308°), 4869 unique (R_{int} = 0.0315, R_{sigma} = 0.0178) which were used in all calculations. The final R_1 was 0.0168 (I > 2σ(I)) and wR_2 was 0.0397 (all data).

Refinement model description

Number of restraints - 0, number of constraints - unknown.

Details:

```
1. Fixed Uiso
 At 1.2 times of:
  All C(H) groups, All N(H,H) groups
 At 1.5 times of:
  All C(H,H,H) groups, All O(H,H) groups
2.a Free rotating group:
 O1W(H1WA,H1WB)
2.b Ternary CH refined with riding coordinates:
 C2(H2), C3(H3), C7(H7), C8(H8)
2.c Secondary CH2 refined with riding coordinates:
 N1(H1A,H1B), N2(H2A,H2B)
2.d Idealised Me refined as rotating group:
 C4(H4A,H4B,H4C), C5(H5A,H5B,H5C), C9(H9A,H9B,H9C), C10(H10A,H10B,H10C)
```

C.Complex (4) *cis*-bis-(isoleucinato)palladium(II)

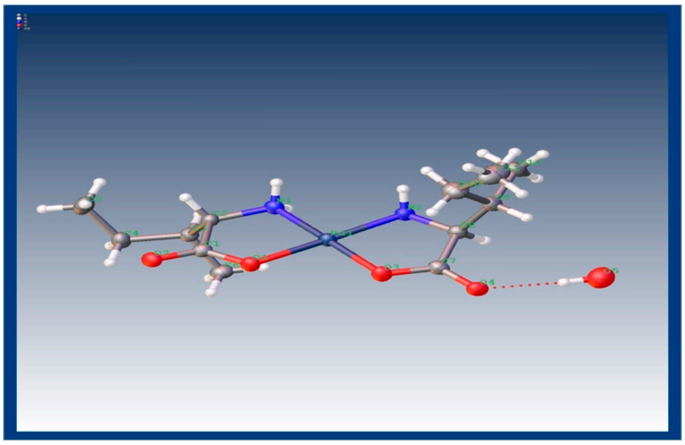

Table S1. Crystal data and structure refinement for Pdisoleucine.

Identification code	Pdisoleucine
Empirical formula	$C_{12}H_{25}N_2O_{4.5}Pd$
Formula weight	375.74
Temperature/K	293(2)
Crystal system	orthorhombic
Space group	$P2_12_12_1$
a/Å	7.5553(2)
b/Å	9.3947(3)
c/Å	21.0623(5)
α/°	90
β/°	90
γ/°	90
Volume/Å^3	1495.00(7)
Z	4
ρ_{calc}g/cm^3	1.669
μ/mm^{-1}	1.257
F(000)	772.0
Crystal size/mm^3	0.374 × 0.291 × 0.205
Radiation	MoKα (λ = 0.71073)
2Θ range for data collection/°	8.678 to 64.178
Index ranges	$-11 \leq h \leq 11$, $-13 \leq k \leq 13$, $-29 \leq l \leq 30$
Reflections collected	32181
Independent reflections	4891 [R_{int} = 0.0327, R_{sigma} = 0.0217]
Data/restraints/parameters	4891/0/188
Goodness-of-fit on F^2	1.095
Final R indexes [I>=2σ (I)]	R_1 = 0.0321, wR_2 = 0.0795
Final R indexes [all data]	R_1 = 0.0356, wR_2 = 0.0821
Largest diff. peak/hole / e Å^{-3}	1.80/-1.47
Flack parameter	-0.044(10)

Table S2. Fractional Atomic Coordinates (×10^4) and Equivalent Isotropic Displacement Parameters (Å^2×10^3) for Pdisoleucine. U_{eq} is defined as 1/3 of of the trace of the orthogonalised U_{IJ} tensor.

Atom	*x*	*y*	*z*	U(eq)
Pd1	2227.0(3)	8161.0(2)	7512.6(2)	19.50(8)
O1	3499(4)	9604(3)	6986.9(13)	24.3(5)
O2	6013(4)	10025(3)	6480.6(13)	25.7(5)

Atom	*x*	*y*	*z*	U(eq)
O3	58(3)	9346(2)	7686.5(13)	25.3(6)
O4	-2646(4)	9245(3)	8093.4(12)	26.7(5)
N1	4327(4)	6938(3)	7307.9(14)	23.0(5)
N2	985(4)	6737(3)	8066.8(14)	22.2(5)
C1	5013(4)	9215(3)	6756.5(17)	21.5(6)
C2	5562(5)	7650(4)	6851.3(16)	21.2(6)
C3	5658(5)	6813(4)	6228.4(16)	22.0(6)
C4	7174(5)	7291(4)	5799.1(18)	27.9(7)
C5	8982(6)	7389(5)	6115(2)	34.8(9)
C6	3903(5)	6825(4)	5870.0(18)	28.5(7)
C7	-1171(5)	8722(4)	8009.8(17)	23.0(6)
C8	-743(5)	7283(4)	8307.0(16)	21.5(6)
C9	-829(6)	7381(4)	9035.0(17)	25.6(8)
C10	446(7)	8495(5)	9305(2)	36.9(9)
C11	-50(7)	8960(7)	9968(2)	49.4(13)
C12	-553(6)	5917(4)	9338.5(19)	32.4(8)
O5	-5383(11)	7978(9)	8784(4)	50.1(17)

Table S3. Anisotropic Displacement Parameters (Å^2×10^3) for Pdisoleucine. The Anisotropic displacement factor exponent takes the form: $-2\pi^2[h^2a^{*2}U_{11}+2hka^*b^*U_{12}+\ldots]$.

Atom	U_{11}	U_{22}	U_{33}	U_{23}	U_{13}	U_{12}
Pd1	21.65(12)	14.06(11)	22.79(12)	-0.52(11)	2.01(11)	0.40(6)
O1	23.6(12)	17.6(11)	31.7(13)	3.1(9)	4.1(10)	0.6(9)
O2	26.2(13)	20.2(11)	30.7(13)	2.8(9)	0.8(10)	-3.7(10)
O3	26.3(13)	17.0(11)	32.6(14)	0.9(9)	5.7(9)	3.4(10)
O4	27.1(13)	23.4(12)	29.7(12)	1.8(10)	3.4(10)	5.3(10)
N1	25.1(14)	17.7(12)	26.1(13)	1.0(10)	2.1(10)	-0.1(11)
N2	23.9(14)	18.7(13)	24.0(13)	-0.2(11)	2.5(11)	2.9(11)
C1	23.0(16)	18.6(15)	22.8(16)	-3.3(11)	-1.7(11)	-0.9(12)
C2	18.9(14)	19.6(14)	25.2(16)	1.1(12)	0.9(12)	0.3(12)
C3	22.6(15)	19.6(14)	23.8(15)	-1.7(12)	0.9(12)	-2.1(12)
C4	27.3(17)	25.1(17)	31.4(17)	0.6(13)	7.6(14)	1.1(14)
C5	23.3(18)	27.5(19)	54(3)	1.7(18)	7.4(18)	2.4(16)
C6	27.9(17)	29.9(18)	27.6(16)	-3.2(14)	-4.5(13)	-1.3(15)
C7	29.1(17)	17.3(14)	22.5(16)	-2.0(12)	0.5(13)	0.2(12)
C8	23.6(15)	17.0(14)	24.0(15)	-1.8(11)	1.6(12)	-0.4(12)
C9	29.0(19)	24.3(17)	23.4(16)	-1.2(13)	5.1(14)	2.2(15)
C10	44(2)	36(2)	29.9(19)	-6.1(16)	-3.8(18)	-4.6(18)
C11	45(3)	62(3)	42(3)	-25(2)	-6.8(19)	9(2)
C12	38(2)	29.2(19)	30.1(18)	8.3(15)	5.6(16)	3.2(16)
O5	42(4)	53(4)	55(4)	-2(3)	5(3)	-1(3)

Table S4. Bond Lengths for Pdisoleucine.

Atom	Atom	Length/Å	Atom	Atom	Length/Å
Pd1	O1	1.997(3)	C1	C2	1.540(5)
Pd1	O3	2.014(2)	C2	C3	1.531(5)
Pd1	N1	2.006(3)	C3	C4	1.527(5)
Pd1	N2	2.008(3)	C3	C6	1.526(5)
O1	C1	1.295(4)	C4	C5	1.521(6)
O2	C1	1.220(4)	C7	C8	1.525(5)
O3	C7	1.292(4)	C8	C9	1.538(5)
O4	C7	1.231(5)	C9	C10	1.532(6)
N1	C2	1.498(5)	C9	C12	1.532(5)
N2	C8	1.492(5)	C10	C11	1.511(6)

Table S5. Bond Angles for Pdisoleucine.

Atom	Atom	Atom	Angle/°	Atom	Atom	Atom	Angle/°
O1	Pd1	O3	96.74(10)	C3	C2	C1	113.1(3)
O1	Pd1	N1	83.61(11)	C4	C3	C2	113.1(3)
O1	Pd1	N2	178.13(12)	C6	C3	C2	112.3(3)
N1	Pd1	O3	177.43(12)	C6	C3	C4	110.9(3)
N1	Pd1	N2	96.49(12)	C5	C4	C3	115.6(3)
N2	Pd1	O3	83.24(11)	O3	C7	C8	117.8(3)
C1	O1	Pd1	116.2(2)	O4	C7	O3	123.0(3)
C7	O3	Pd1	115.5(2)	O4	C7	C8	119.2(3)
C2	N1	Pd1	112.0(2)	N2	C8	C7	110.6(3)
C8	N2	Pd1	112.1(2)	N2	C8	C9	113.3(3)
O1	C1	C2	117.3(3)	C7	C8	C9	110.3(3)
O2	C1	O1	123.3(3)	C10	C9	C8	112.6(3)
O2	C1	C2	119.4(3)	C12	C9	C8	110.9(3)
N1	C2	C1	110.0(3)	C12	C9	C10	111.9(4)
N1	C2	C3	110.5(3)	C11	C10	C9	112.6(4)

Table S6. Hydrogen Bonds for Pdisoleucine.

D	H	A	d(D-H)/Å	d(H-A)/Å	d(D-A)/Å	D-H-A/°
N1	H1B	O4[1]	0.89	2.08	2.954(4)	167.1
N2	H2A	O3[1]	0.89	1.98	2.861(4)	168.8
N2	H2B	O2[2]	0.89	2.26	2.939(4)	133.3
O5	H5D	O4	0.85	1.95	2.795(8)	170.4
O5	H5E	O2[1]	0.85	2.08	2.869(8)	155.1

[1]-X,-1/2+Y,3/2-Z; [2]1-X,-1/2+Y,3/2-Z.

Table S7. Torsion Angles for Pdisoleucine.

A	B	C	D	Angle/°	A	B	C	D	Angle/°
Pd1	O1	C1	O2	-172.6(3)	O4	C7	C8	N2	171.0(3)
Pd1	O1	C1	C2	6.4(4)	O4	C7	C8	C9	-62.8(4)
Pd1	O3	C7	O4	-171.7(3)	N1	C2	C3	C4	-167.7(3)
Pd1	O3	C7	C8	9.3(4)	N1	C2	C3	C6	66.0(4)
Pd1	N1	C2	C1	9.3(3)	N2	C8	C9	C10	66.2(4)
Pd1	N1	C2	C3	-116.3(3)	N2	C8	C9	C12	-60.1(4)
Pd1	N2	C8	C7	5.6(3)	C1	C2	C3	C4	68.6(4)
Pd1	N2	C8	C9	-118.8(3)	C1	C2	C3	C6	-57.8(4)
O1	C1	C2	N1	-10.4(4)	C2	C3	C4	C5	50.1(4)
O1	C1	C2	C3	113.6(3)	C6	C3	C4	C5	177.2(3)
O2	C1	C2	N1	168.6(3)	C7	C8	C9	C10	-58.4(4)
O2	C1	C2	C3	-67.3(4)	C7	C8	C9	C12	175.3(3)
O3	C7	C8	N2	-9.9(4)	C8	C9	C10	C11	160.3(4)
O3	C7	C8	C9	116.2(4)	C12	C9	C10	C11	-74.0(5)

Table S8. Hydrogen Atom Coordinates (Å×10[4]) and Isotropic Displacement Parameters (Å[2]×10[3]) for Pdisoleucine.

Atom	*x*	*y*	*z*	U(eq)
H1A	4907.73	6739.09	7664.62	28
H1B	3954.69	6120.93	7141.2	28
H2A	800.76	5942.21	7846.36	27
H2B	1676.57	6521.8	8395.23	27
H2	6746.14	7644.23	7041.32	25
H3	5895.93	5819.7	6342.43	26
H4A	7257.9	6632.08	5445.85	34
H4B	6880.91	8218.27	5626.1	34
H5A	9881.05	7468.44	5794.82	52
H5B	9186.1	6548.1	6363.43	52
H5C	9017.85	8210.57	6385.18	52
H6A	2977.42	6489.7	6144.08	43
H6B	3987.72	6214.65	5505.65	43

Atom	*x*	*y*	*z*	U(eq)
H6C	3640.5	7777.49	5734.85	43
H8	-1662.18	6612.56	8171.17	26
H9	-2029.19	7689.57	9145.13	31
H10A	1633.58	8102.13	9311.24	44
H10B	455	9319.96	9027.99	44
H11A	-1227.35	9343.44	9965.65	74
H11B	765.24	9675.39	10111.15	74
H11C	-2.08	8156.18	10249.13	74
H12A	-1333.47	5239.33	9143.45	49
H12B	-803.7	5971.68	9784.67	49
H12C	651.52	5620.86	9277.71	49
H5D	-4602.4	8456.44	8586.92	75
H5E	-5346.66	7158.25	8613.83	75

Table S9. Atomic Occupancy for Pdisoleucine.

Atom	*Occupancy*	Atom	*Occupancy*	Atom	*Occupancy*
O5	0.5	H5D	0.5	H5E	0.5

Experimental

Single crystals of $C_{12}H_{25}N_2O_{4.5}Pd$ [Pdisoleucine] were **grown by allowing an acetone/water solution to evaporate slowly** . A suitable crystal was selected and mounted on a fiber on a **Xcalibur, Sapphire3, Gemini ultra** diffractometer. The crystal was kept at 293(2) K during data collection. Using Olex2 [1], the structure was solved with the SHELXT [2] structure solution program using Intrinsic Phasing and refined with the SHELXL [3] refinement package using CGLS minimisation.

1. Dolomanov, O.V., Bourhis, L.J., Gildea, R.J, Howard, J.A.K. & Puschmann, H. (2009), J. Appl. Cryst. 42, 339-341.
2. Sheldrick, G.M. (2015). Acta Cryst. A71, 3-8.
3. Sheldrick, G.M. (2015). Acta Cryst. C71, 3-8.

Crystal structure determination of [Pdisoleucine]

Crystal Data for $C_{12}H_{25}N_2O_{4.5}Pd$ (M =375.74 g/mol): orthorhombic, space group $P2_12_12_1$ (no. 19), a = 7.5553(2) Å, b = 9.3947(3) Å, c = 21.0623(5) Å, V = 1495.00(7) Å^3, Z = 4, T = 293(2) K, μ(MoKα) = 1.257 mm^{-1}, $Dcalc$ = 1.669 g/cm^3, 32181 reflections measured (8.678° ≤ 2Θ ≤ 64.178°), 4891 unique (R_{int} = 0.0327, R_{sigma} = 0.0217) which were used in all calculations. The final R_1 was 0.0321 (I > 2σ(I)) and wR_2 was 0.0821 (all data).

Refinement model description

Number of restraints - 0, number of constraints - unknown.

Details:

```
1. Fixed Uiso
 At 1.2 times of:
  All C(H) groups, All C(H,H) groups, All N(H,H) groups
 At 1.5 times of:
  All C(H,H,H) groups, All O(H,H) groups
2. Others
 Fixed Sof: O5(0.5) H5D(0.5) H5E(0.5)
3.a Free rotating group:
 O5(H5D,H5E)
3.b Ternary CH refined with riding coordinates:
 C2(H2), C3(H3), C8(H8), C9(H9)
3.c Secondary CH2 refined with riding coordinates:
 N1(H1A,H1B), N2(H2A,H2B), C4(H4A,H4B), C10(H10A,H10B)
3.d Idealised Me refined as rotating group:
 C5(H5A,H5B,H5C), C6(H6A,H6B,H6C), C11(H11A,H11B,H11C), C12(H12A,H12B,H12C)
```

D.Complex (15) *cis*-bis-(serinato)palladium(II)

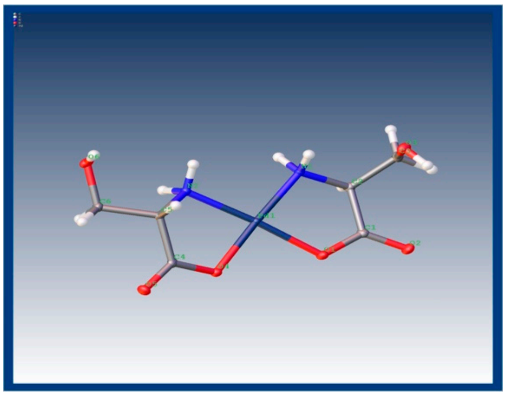

Table S1. Crystal data and structure refinement for Pdserine.

Identification code	Pdserine
Empirical formula	$C_6H_{12}N_2O_6Pd$
Formula weight	314.58
Temperature/K	100.05(10)
Crystal system	orthorhombic
Space group	$P2_12_12_1$
a/Å	8.71610(10)
b/Å	9.67670(10)
c/Å	11.2446(2)
α/°	90
β/°	90
γ/°	90
Volume/Å^3	948.40(2)
Z	4
ρ_{calc}g/cm^3	2.203
μ/mm^{-1}	1.970
F(000)	624.0
Crystal size/mm^3	0.411 × 0.253 × 0.189
Radiation	Mo Kα (λ = 0.71073)
2Θ range for data collection/°	7.248 to 65.028
Index ranges	$-12 \le h \le 12$, $-14 \le k \le 14$, $-16 \le l \le 16$
Reflections collected	20421
Independent reflections	3260 [R_{int} = 0.0329, R_{sigma} = 0.0200]
Data/restraints/parameters	3260/0/143
Goodness-of-fit on F^2	1.091
Final R indexes [I>=2σ (I)]	R_1 = 0.0148, wR_2 = 0.0346
Final R indexes [all data]	R_1 = 0.0156, wR_2 = 0.0350
Largest diff. peak/hole / e Å^{-3}	0.55/-0.50
Flack parameter	-0.047(11)

Table S2. Fractional Atomic Coordinates (×10^4) and Equivalent Isotropic Displacement Parameters (Å^2×10^3) for Pdserine. U_{eq} is defined as 1/3 of of the trace of the orthogonalised U_{IJ} tensor.

Atom	*x*	*y*	*z*	U(eq)
Pd1	3878.8(2)	5821.0(2)	5482.8(2)	6.98(5)
O1	3187.1(19)	4721.6(16)	6888.2(14)	11.2(3)
O2	3496.6(19)	2713.3(16)	7810.0(15)	14.5(3)
O3	7053.8(18)	2623.4(18)	6432.1(15)	13.1(3)
O4	2889.4(17)	7588.0(17)	6047.3(13)	9.5(3)
O5	2820.3(19)	9851.0(16)	5705.5(14)	12.4(3)
O6	5321.1(17)	9232(2)	2512.5(13)	12.7(3)

Atom	*x*	*y*	*z*	U(eq)
N1	4898(2)	4038.1(19)	5012.2(16)	9.5(3)
N2	4440(2)	7014.1(19)	4077.5(17)	9.0(3)
C1	3679(2)	3448(2)	6934.6(19)	9.2(4)
C2	4409(3)	2889(2)	5800.5(19)	8.7(4)
C3	5732(2)	1897(2)	6038(2)	11.0(4)
C4	3307(2)	8697(2)	5462(2)	8.4(3)
C5	4482(2)	8481.1(19)	4475(2)	8.3(3)
C6	4245(2)	9486(2)	3443.5(19)	10.4(4)

Table S3. Anisotropic Displacement Parameters (Å^2×10^3) for Pdserine. The Anisotropic displacement factor exponent takes the form: $-2\pi^2[h^2a^{*2}U_{11}+2hka^*b^*U_{12}+\ldots]$.

Atom	U_{11}	U_{22}	U_{33}	U_{23}	U_{13}	U_{12}
Pd1	7.89(6)	6.69(6)	6.35(6)	0.28(5)	0.61(5)	0.55(5)
O1	12.9(7)	9.9(7)	10.7(7)	1.0(6)	3.6(6)	1.0(6)
O2	17.1(8)	13.6(8)	12.8(7)	4.3(6)	4.8(6)	0.4(6)
O3	10.7(6)	18.9(8)	9.7(7)	1.5(6)	-1.4(6)	-1.6(6)
O4	10.0(6)	9.0(7)	9.5(7)	0.2(6)	1.8(5)	0.2(6)
O5	14.2(7)	8.8(7)	14.3(8)	-0.1(5)	1.8(6)	2.5(6)
O6	11.2(7)	18.6(7)	8.2(6)	1.1(7)	1.0(5)	-1.3(7)
N1	11.9(7)	9.2(8)	7.5(7)	0.6(7)	1.5(6)	1.3(7)
N2	10.7(7)	8.4(7)	8.0(8)	0.3(6)	0.6(6)	1.2(6)
C1	7.5(9)	9.4(8)	10.8(9)	0.0(7)	0.8(7)	-1.3(7)
C2	9.6(8)	7.8(8)	8.7(9)	0.8(6)	-0.3(7)	-1.1(7)
C3	12.4(9)	9.7(9)	10.8(9)	0.3(7)	-0.9(7)	1.1(7)
C4	7.0(7)	10.2(8)	8.0(8)	1.3(8)	-1.8(8)	-0.9(6)
C5	9.0(8)	8.1(8)	7.7(8)	0.2(7)	0.3(8)	0.2(6)
C6	11.4(9)	10.5(9)	9.2(9)	1.9(7)	1.0(7)	0.9(6)

Table S4 Bond Lengths for Pdserine.

Atom	Atom	Length/Å	Atom	Atom	Length/Å
Pd1	O1	1.9981(16)	O5	C4	1.226(2)
Pd1	O4	2.0175(16)	O6	C6	1.427(2)
Pd1	N1	2.0114(18)	N1	C2	1.485(3)
Pd1	N2	2.0173(19)	N2	C5	1.489(3)
O1	C1	1.306(2)	C1	C2	1.525(3)
O2	C1	1.225(3)	C2	C3	1.524(3)
O3	C3	1.421(3)	C4	C5	1.524(3)
O4	C4	1.310(3)	C5	C6	1.528(3)

Table S5 Bond Angles for Pdserine.

Atom	Atom	Atom	Angle/°	Atom	Atom	Atom	Angle/°
O1	Pd1	O4	94.21(6)	O2	C1	C2	121.36(18)
O1	Pd1	N1	83.38(7)	N1	C2	C1	110.68(17)
O1	Pd1	N2	175.89(7)	N1	C2	C3	111.05(17)
N1	Pd1	O4	176.88(7)	C3	C2	C1	113.13(17)
N1	Pd1	N2	100.23(7)	O3	C3	C2	110.92(17)
N2	Pd1	O4	82.25(6)	O4	C4	C5	116.15(17)
C1	O1	Pd1	115.79(14)	O5	C4	O4	122.5(2)
C4	O4	Pd1	114.63(13)	O5	C4	C5	121.33(18)
C2	N1	Pd1	111.02(13)	N2	C5	C4	109.41(16)
C5	N2	Pd1	108.47(13)	N2	C5	C6	112.11(18)
O1	C1	C2	116.02(18)	C4	C5	C6	112.00(16)
O2	C1	O1	122.5(2)	O6	C6	C5	110.99(16)

Table S6 Hydrogen Bonds for Pdserine.

D	H	A	d(D-H)/Å	d(H-A)/Å	d(D-A)/Å	D-H-A/°
O3	H3	O4[1]	0.83(3)	2.01(3)	2.835(2)	178(3)
O6	H6	O1[2]	0.81(3)	1.96(3)	2.778(2)	176(3)
N1	H1A	O6[3]	0.91	2.02	2.852(2)	152.1
N1	H1B	O5[2]	0.91	2.10	2.880(2)	143.8

[1]1-X,-1/2+Y,3/2-Z; [2]1/2+X,3/2-Y,1-Z; [3]1-X,-1/2+Y,1/2-Z.

Table S7. Torsion Angles for Pdserine.

A	B	C	D	Angle/°	A	B	C	D	Angle/°
Pd1	O1	C1	O2	171.09(17)	O2	C1	C2	N1	-164.06(19)
Pd1	O1	C1	C2	-12.7(2)	O2	C1	C2	C3	-38.7(3)
Pd1	O4	C4	O5	-179.23(17)	O4	C4	C5	N2	22.3(2)
Pd1	O4	C4	C5	-1.1(2)	O4	C4	C5	C6	147.27(18)
Pd1	N1	C2	C1	-16.9(2)	O5	C4	C5	N2	-159.5(2)
Pd1	N1	C2	C3	-143.40(14)	O5	C4	C5	C6	-34.6(3)
Pd1	N2	C5	C4	-31.80(19)	N1	C2	C3	O3	51.3(2)
Pd1	N2	C5	C6	-156.68(13)	N2	C5	C6	O6	-54.4(2)
O1	C1	C2	N1	19.7(2)	C1	C2	C3	O3	-73.9(2)
O1	C1	C2	C3	145.05(19)	C4	C5	C6	O6	-177.87(17)

Table S8. Hydrogen Atom Coordinates (Å×10^4) and Isotropic Displacement Parameters (Å^2×10^3) for Pdserine.

Atom	*x*	*y*	*z*	U(eq)
H3	7040(40)	2610(30)	7170(30)	20
H6	6150(40)	9530(30)	2720(30)	19
H1A	4650.93	3831.82	4246.2	11
H1B	5934.52	4138.53	5055.97	11
H2A	5374.42	6760.57	3789.1	11
H2B	3734.47	6908.48	3488.26	11
H2	3599.33	2357.69	5366.42	10
H3A	5421.05	1218.09	6651.1	13
H3B	5978.3	1384.16	5300.72	13
H5	5521.81	8661.33	4817.44	10
H6A	3189.49	9385.35	3130.38	12
H6B	4367.01	10446.12	3733.62	12

Experimental

Single crystals of $C_6H_{12}N_2O_6Pd$ **[Pdserine]** were **grown by allowing an acetone/water solution to evaporate slowly**. A suitable crystal was selected and and mounted on fiber on a **Xcalibur, Sapphire3, Gemini ultra** diffractometer. The crystal was kept at 100.05(10) K during data collection. Using Olex2 [1], the structure was solved with the SHELXT [2] structure solution program using Intrinsic Phasing and refined with the SHELXL [3] refinement package using Least Squares minimisation.

1. Dolomanov, O.V., Bourhis, L.J., Gildea, R.J, Howard, J.A.K. & Puschmann, H. (2009), J. Appl. Cryst. 42, 339-341.
2. Sheldrick, G.M. (2015). Acta Cryst. A71, 3-8.
3. Sheldrick, G.M. (2015). Acta Cryst. C71, 3-8.

Crystal structure determination of [Pdserine]

Crystal Data for $C_6H_{12}N_2O_6Pd$ (M =314.58 g/mol): orthorhombic, space group $P2_12_12_1$ (no. 19), a = 8.71610(10) Å, b = 9.67670(10) Å, c = 11.2446(2) Å, V = 948.40(2) Å^3, Z = 4, T = 100.05(10) K, μ(Mo Kα) = 1.970 mm^{-1}, $Dcalc$ = 2.203 g/cm^3, 20421 reflections measured (7.248° ≤ 2Θ ≤ 65.028°), 3260 unique (R_{int} = 0.0329, R_{sigma} = 0.0200) which were used in all calculations. The final R_1 was 0.0148 (I > 2σ(I)) and wR_2 was 0.0350 (all data).

Refinement model description

Number of restraints - 0, number of constraints - unknown.

Details:

```
1. Fixed Uiso
 At 1.2 times of:
  All C(H) groups, All C(H,H) groups, All N(H,H) groups
 At 1.5 times of:
  All O(H) groups
```

```
2.a Ternary CH refined with riding coordinates:
 C2(H2), C5(H5)
2.b Secondary CH2 refined with riding coordinates:
 N1(H1A,H1B), N2(H2A,H2B), C3(H3A,H3B), C6(H6A,H6B)
```

E. Complex (16) *cis*-bis(threoninato)palladium(II)

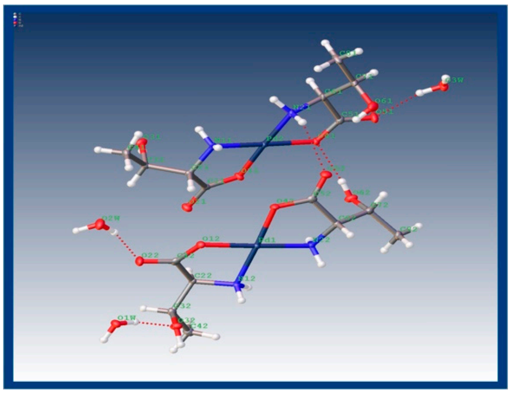

Table S1. Crystal data and structure refinement for threoninePd.

Identification code	threoninePd
Empirical formula	$C_{16}H_{38}N_4O_{15}Pd_2$
Formula weight	739.30
Temperature/K	100.00(10)
Crystal system	orthorhombic
Space group	$P2_12_12_1$
a/Å	10.6869(2)
b/Å	11.3234(3)
c/Å	21.0572(3)
α/°	90
β/°	90
γ/°	90
Volume/Å^3	2548.17(9)
Z	4
ρ_{calc}g/cm^3	1.927
μ/mm^{-1}	1.490
F(000)	1496.0
Crystal size/mm^3	0.418 × 0.264 × 0.259
Radiation	Mo Kα (λ = 0.71073)
2Θ range for data collection/°	6.516 to 65.618
Index ranges	$-15 \le h \le 15$, $-13 \le k \le 16$, $-30 \le l \le 31$
Reflections collected	22934
Independent reflections	8597 [R_{int} = 0.0555, R_{sigma} = 0.0814]
Data/restraints/parameters	8597/0/351
Goodness-of-fit on F^2	1.003
Final R indexes [I>=2σ (I)]	R_1 = 0.0404, wR_2 = 0.0632
Final R indexes [all data]	R_1 = 0.0569, wR_2 = 0.0691
Largest diff. peak/hole / e Å^{-3}	0.74/-1.10
Flack parameter	-0.04(2)

Table S2. Fractional Atomic Coordinates (×10^4) and Equivalent Isotropic Displacement Parameters (Å^2×10^3) for threoninePd. U_{eq} is defined as 1/3 of of the trace of the orthogonalised U_{IJ} tensor.

Atom	x	y	z	U(eq)
Pd1	2219.6(3)	2543.5(3)	3494.7(2)	8.49(7)
O12	1389(3)	3130(3)	2688.6(14)	10.5(7)
O22	1014(3)	2578(3)	1690.6(14)	17.5(8)
O32	-196(3)	306(3)	2259.9(15)	13.5(7)
O42	2579(3)	4154(3)	3854.5(15)	11.8(7)
O52	3560(3)	5052(3)	4656.1(16)	15.7(8)
O62	5466(3)	2680(3)	4763.9(16)	15.4(8)
N12	1847(4)	960(4)	3101.4(17)	10.4(8)
N22	3126(3)	1976(4)	4272.6(16)	8.9(8)
C12	1363(4)	2347(5)	2233(2)	12.1(10)
C22	1827(4)	1109(4)	2403(2)	10.2(10)
C32	1088(4)	144(5)	2071(2)	12.5(10)
C42	1545(4)	-1084(5)	2236(2)	15.7(11)
C52	3151(4)	4145(5)	4406(2)	11.9(10)
C62	3218(4)	2962(4)	4745(2)	10.1(10)
C72	4380(4)	2816(4)	5159(2)	13.1(11)
C82	4299(5)	1725(5)	5577(2)	15.5(11)
Pd2	5291.0(3)	5327.9(3)	3516.0(2)	8.36(7)
O11	5259(3)	3813(3)	3019.0(14)	12.1(7)
O21	4337(3)	2939(3)	2198.2(15)	14.3(8)
O31	5148(3)	5458(3)	1531.6(16)	14.7(7)
O41	6399(3)	4746(3)	4229.7(13)	11.0(7)
O51	7282(3)	5312(3)	5134.6(14)	18.3(8)
O61	5248(4)	7168(3)	5398.0(15)	22.3(9)
N11	4206(4)	5924(3)	2805.1(16)	8.9(8)
N21	5294(4)	6808(4)	4045.0(16)	12.2(8)
C11	4501(4)	3807(4)	2541(2)	10.1(10)
C21	3775(4)	4932(4)	2400(2)	9.8(10)
C31	3863(4)	5233(5)	1694.1(19)	11.3(10)
C41	3105(4)	6300(4)	1504(2)	15.6(10)
C51	6724(4)	5554(5)	4640(2)	12.9(10)
C61	6415(4)	6829(5)	4460(2)	13.0(10)
C71	6243(5)	7647(5)	5032(2)	15.6(11)
C81	5976(5)	8918(5)	4858(2)	20.7(12)
O3W	8109(3)	6286(3)	6251.4(15)	15.4(8)
O2W	220(4)	4835(3)	1240.9(15)	21.3(8)
O1W	-1885(3)	1521(3)	1493.1(18)	14.4(7)

Table S3 Anisotropic Displacement Parameters (Å^2×10^3) for threoninePd. The Anisotropic displacement factor exponent takes the form: $-2\pi^2[h^2a^{*2}U_{11}+2hka^*b^*U_{12}+\ldots]$.

Atom	U_{11}	U_{22}	U_{33}	U_{23}	U_{13}	U_{12}
Pd1	9.20(14)	7.99(18)	8.27(12)	0.06(15)	-0.61(14)	-0.12(14)
O12	11.1(16)	8.5(18)	11.8(14)	0.4(13)	-1.9(12)	1.2(14)
O22	26.6(19)	15(2)	10.9(13)	2.2(15)	-4.9(14)	1.1(18)
O32	9.3(15)	11.6(19)	19.5(15)	2.1(15)	-0.7(14)	-0.4(16)
O42	14.8(17)	7.1(18)	13.5(14)	0.6(13)	-1.1(13)	-1.8(14)
O52	19.1(18)	11(2)	17.1(16)	-2.7(14)	0.3(15)	-1.9(15)
O62	11.4(17)	12(2)	22.2(16)	3.7(15)	2.3(14)	0.1(16)
N12	14(2)	6(2)	11.0(16)	-0.3(15)	-3.3(15)	-0.2(17)
N22	8.4(18)	10(2)	8.8(15)	0.0(15)	-1.3(14)	-1.8(16)
C12	8(2)	15(3)	13.1(18)	2.8(19)	1.7(16)	0(2)
C22	11(2)	10(3)	9.4(18)	2.3(17)	-2.4(17)	-3(2)
C32	11(2)	16(3)	10.6(18)	-1.0(18)	0.2(17)	-1(2)
C42	14(2)	17(3)	16(2)	-3(2)	0.9(19)	1(2)
C52	8(2)	14(3)	14(2)	-0.3(19)	2.1(17)	1(2)
C62	11(2)	10(3)	8.6(18)	-0.9(17)	-0.3(17)	0(2)
C72	14(2)	14(3)	11.5(19)	1.2(18)	-0.5(17)	-2(2)
C82	18(2)	19(3)	9.8(19)	1(2)	-1.4(18)	-1(2)

Atom	U_{11}	U_{22}	U_{33}	U_{23}	U_{13}	U_{12}
Pd2	10.40(14)	8.22(18)	6.47(11)	-0.11(14)	-0.39(14)	0.38(14)
O11	13.1(16)	11.2(19)	12.1(14)	-3.3(13)	-3.8(14)	0.6(16)
O21	15.9(18)	12(2)	14.7(15)	-3.3(13)	-3.5(13)	1.8(15)
O31	16.3(15)	13.8(18)	14.1(13)	0.1(15)	6.7(15)	0.7(14)
O41	14.3(16)	11.2(19)	7.4(12)	-0.5(13)	-1.8(12)	0.2(15)
O51	29(2)	15(2)	10.9(13)	-1.8(14)	-7.5(15)	4.1(19)
O61	35(2)	20(2)	12.0(14)	-1.7(14)	4.1(16)	-2.8(19)
N11	12.0(19)	9(2)	6.0(15)	0.1(14)	1.2(14)	1.7(17)
N21	15.2(19)	12(2)	9.0(15)	-1.4(15)	-3.3(16)	1.1(19)
C11	8(2)	11(3)	11.5(18)	-0.5(17)	2.0(17)	-2(2)
C21	10(2)	9(3)	10.1(18)	-0.7(17)	1.7(16)	-1.7(19)
C31	14(2)	11(3)	8.8(16)	-2.6(18)	0.2(16)	-2(2)
C41	19(2)	15(3)	12.7(18)	1(2)	-5(2)	-1(2)
C51	14(2)	14(3)	10.4(19)	0.6(18)	0.7(17)	2(2)
C61	16(2)	16(3)	7.7(17)	1.0(19)	-3.7(17)	-3(2)
C71	26(3)	10(3)	10.5(19)	0.6(18)	-7.6(18)	-1(2)
C81	32(3)	13(3)	18(2)	-5(2)	-10(2)	3(3)
O3W	18.8(19)	16(2)	11.6(14)	-1.9(14)	-3.3(14)	5.3(16)
O2W	34(2)	16(2)	13.5(14)	2.8(14)	-1.8(16)	4.6(19)
O1W	17.7(17)	11.2(18)	14.3(14)	-0.8(16)	-4.4(16)	-0.1(14)

Table S4. Bond Lengths for threoninePd.

Atom	Atom	Length/Å	Atom	Atom	Length/Å
Pd1	O12	2.027(3)	Pd2	O11	2.009(3)
Pd1	O42	2.012(3)	Pd2	O41	2.023(3)
Pd1	N12	2.015(4)	Pd2	N11	2.010(4)
Pd1	N22	2.009(4)	Pd2	N21	2.013(4)
O12	C12	1.307(6)	O11	C11	1.292(5)
O22	C12	1.230(5)	O21	C11	1.232(5)
O32	C32	1.440(5)	O31	C31	1.439(5)
O42	C52	1.312(5)	O41	C51	1.305(6)
O52	C52	1.233(6)	O51	C51	1.231(5)
O62	C72	1.435(5)	O61	C71	1.421(6)
N12	C22	1.481(5)	N11	C21	1.484(6)
N22	C62	1.498(6)	N21	C61	1.484(6)
C12	C22	1.528(7)	C11	C21	1.521(7)
C22	C32	1.518(7)	C21	C31	1.528(6)
C32	C42	1.514(7)	C31	C41	1.509(7)
C52	C62	1.519(7)	C51	C61	1.528(7)
C62	C72	1.526(6)	C61	C71	1.530(7)
C72	C82	1.520(7)	C71	C81	1.512(7)

Table S5. Bond Angles for threoninePd.

Atom	Atom	Atom	Angle/°	Atom	Atom	Atom	Angle/°
O42	Pd1	O12	95.88(13)	O11	Pd2	O41	96.85(13)
O42	Pd1	N12	177.78(14)	O11	Pd2	N11	83.62(14)
N12	Pd1	O12	81.98(15)	O11	Pd2	N21	177.61(14)
N22	Pd1	O12	177.13(14)	N11	Pd2	O41	179.23(16)
N22	Pd1	O42	83.73(15)	N11	Pd2	N21	97.66(15)
N22	Pd1	N12	98.39(16)	N21	Pd2	O41	81.89(14)
C12	O12	Pd1	113.7(3)	C11	O11	Pd2	114.9(3)
C52	O42	Pd1	114.6(3)	C51	O41	Pd2	114.8(3)
C22	N12	Pd1	108.0(3)	C21	N11	Pd2	110.7(3)
C62	N22	Pd1	109.5(3)	C61	N21	Pd2	109.9(3)
O12	C12	C22	116.3(4)	O11	C11	C21	117.8(4)
O22	C12	O12	122.9(5)	O21	C11	O11	123.4(4)
O22	C12	C22	120.7(4)	O21	C11	C21	118.8(4)

Atom	Atom	Atom	Angle/°	Atom	Atom	Atom	Angle/°
N12	C22	C12	110.0(4)	N11	C21	C11	111.3(4)
N12	C22	C32	112.5(4)	N11	C21	C31	111.8(4)
C32	C22	C12	112.6(4)	C11	C21	C31	110.2(4)
O32	C32	C22	106.1(4)	O31	C31	C21	109.2(4)
O32	C32	C42	111.1(4)	O31	C31	C41	107.9(4)
C42	C32	C22	112.9(4)	C41	C31	C21	113.8(4)
O42	C52	C62	116.3(4)	O41	C51	C61	116.1(4)
O52	C52	O42	122.5(5)	O51	C51	O41	122.2(5)
O52	C52	C62	121.1(4)	O51	C51	C61	121.6(4)
N22	C62	C52	110.1(4)	N21	C61	C51	107.8(4)
N22	C62	C72	110.6(4)	N21	C61	C71	112.1(4)
C52	C62	C72	113.7(4)	C51	C61	C71	113.8(4)
O62	C72	C62	109.8(4)	O61	C71	C61	106.6(4)
O62	C72	C82	107.2(4)	O61	C71	C81	110.7(4)
C82	C72	C62	112.0(4)	C81	C71	C61	114.1(4)

Table S6. Hydrogen Bonds for threoninePd.

D	H	A	d(D-H)/Å	d(H-A)/Å	d(D-A)/Å	D-H-A/°
O32	H32	O12[1]	0.82	1.97	2.777(5)	169.1
O62	H62	O41	0.82	1.97	2.781(5)	171.0
N12	H12A	O3W[2]	0.89	2.37	3.185(5)	152.1
N12	H12B	O2W[1]	0.89	2.04	2.901(5)	161.5
N22	H22A	O51[2]	0.89	2.15	3.014(5)	162.7
N22	H22B	O31[3]	0.89	2.30	3.037(5)	139.9
O31	H31	O3W[4]	0.82	2.00	2.778(5)	158.9
N11	H11A	O1W[5]	0.89	2.12	2.965(5)	157.2
N11	H11B	O21[6]	0.89	2.08	2.762(5)	132.4
N21	H21A	O21[6]	0.89	2.20	2.941(5)	140.8
N21	H21B	O52	0.89	2.44	3.008(5)	122.2
O3W	H3WA	O1W[7]	0.85	2.01	2.853(5)	172.8
O3W	H3WB	O51	0.85	1.92	2.743(5)	163.3
O2W	H2WA	O22	0.85	2.03	2.854(5)	164.7
O2W	H2WB	O61[8]	0.85	2.26	2.923(5)	134.6
O1W	H1WA	O42[1]	0.85	2.08	2.876(5)	155.0
O1W	H1WB	O32	0.85	1.95	2.785(5)	168.4

[1]-X,-1/2+Y,1/2-Z; [2]-1/2+X,1/2-Y,1-Z; [3]1-X,-1/2+Y,1/2-Z; [4]3/2-X,1-Y,-1/2+Z; [5]-X,1/2+Y,1/2-Z; [6]1-X,1/2+Y,1/2-Z; [7]1/2-X,1-Y,1/2+Z; [8]1/2-X,1-Y,-1/2+Z.

Table S7. Torsion Angles for threoninePd.

A	B	C	D	Angle/°	A	B	C	D	Angle/°
Pd1	O12	C12	O22	-172.4(4)	Pd2	O11	C11	O21	-177.7(3)
Pd1	O12	C12	C22	6.1(5)	Pd2	O11	C11	C21	2.1(5)
Pd1	O42	C52	O52	171.8(4)	Pd2	O41	C51	O51	172.1(4)
Pd1	O42	C52	C62	-11.8(5)	Pd2	O41	C51	C61	-10.1(5)
Pd1	N12	C22	C12	-31.6(4)	Pd2	N11	C21	C11	-13.1(4)
Pd1	N12	C22	C32	-157.9(3)	Pd2	N11	C21	C31	-136.8(3)
Pd1	N22	C62	C52	-25.0(4)	Pd2	N21	C61	C51	-32.7(4)
Pd1	N22	C62	C72	-151.5(3)	Pd2	N21	C61	C71	-158.7(3)
O12	C12	C22	N12	17.5(6)	O11	C11	C21	N11	7.5(6)
O12	C12	C22	C32	143.7(4)	O11	C11	C21	C31	132.2(4)
O22	C12	C22	N12	-164.0(4)	O21	C11	C21	N11	-172.7(4)
O22	C12	C22	C32	-37.8(6)	O21	C11	C21	C31	-48.1(6)
O42	C52	C62	N22	24.8(6)	O41	C51	C61	N21	28.6(5)
O42	C52	C62	C72	149.5(4)	O41	C51	C61	C71	153.5(4)
O52	C52	C62	N22	-158.7(4)	O51	C51	C61	N21	-153.6(4)
O52	C52	C62	C72	-34.0(6)	O51	C51	C61	C71	-28.7(7)
N12	C22	C32	O32	66.7(5)	N11	C21	C31	O31	61.6(5)

A	B	C	D	Angle/°	A	B	C	D	Angle/°
N12	C22	C32	C42	-55.2(5)	N11	C21	C31	C41	-59.0(5)
N22	C62	C72	O62	53.1(5)	N21	C61	C71	O61	63.4(5)
N22	C62	C72	C82	-65.8(5)	N21	C61	C71	C81	-59.1(6)
C12	C22	C32	O32	-58.2(5)	C11	C21	C31	O31	-62.7(5)
C12	C22	C32	C42	179.9(4)	C11	C21	C31	C41	176.6(4)
C52	C62	C72	O62	-71.3(5)	C51	C61	C71	O61	-59.2(5)
C52	C62	C72	C82	169.8(4)	C51	C61	C71	C81	178.3(4)

Table S8. Hydrogen Atom Coordinates (Å×10^4) and Isotropic Displacement Parameters (Å^2×10^3) for threoninePd.

Atom	*x*	*y*	*z*	U(eq)
H32	-518.22	-339.95	2325.13	20
H62	5687.59	3328.9	4632.59	23
H12A	2431.86	439.49	3211.1	12
H12B	1109.13	695.13	3236.66	12
H22A	2715.47	1370.56	4443.53	11
H22B	3889.62	1730.52	4166.97	11
H22	2693.38	1049.77	2254.27	12
H32A	1153.36	257.46	1611.02	15
H42A	1412.07	-1229.97	2679.88	24
H42B	2421.19	-1145.52	2141.45	24
H42C	1090.38	-1655.85	1990.56	24
H62A	2487.14	2908.16	5023.9	12
H72	4481.1	3516.82	5426.37	16
H82A	4277.23	1031.33	5315.92	23
H82B	3552.86	1762.46	5830.24	23
H82C	5017.4	1693.77	5850.94	23
H31	5512.65	4828.86	1476.25	22
H61	4701.88	6917.54	5160.09	33
H11A	3546.64	6299.62	2965.85	11
H11B	4637.08	6435.53	2570.2	11
H21A	5297.33	7438.49	3792.34	15
H21B	4605.09	6834.57	4282.09	15
H21	2892.7	4783.94	2499.82	12
H31A	3569.79	4551.58	1448.42	14
H41A	3421.54	6985.81	1718.65	23
H41B	2244.9	6180.33	1618.47	23
H41C	3166.48	6414.17	1053.06	23
H61A	7115.33	7134.58	4208.82	16
H71	7008.39	7623.84	5288.03	19
H81A	5225.14	8956.39	4608.62	31
H81B	6663.14	9229.45	4616.94	31
H81C	5869.94	9375.02	5238.29	31
H3WA	7727.83	6915.47	6355.7	23
H3WB	7838.86	6128.56	5881.19	23
H2WA	543.7	4237.03	1421.75	32
H2WB	78.59	4606.77	862.86	32
H1WA	-2136.96	926.97	1281.52	22
H1WB	-1323.69	1244.8	1737.62	22

Experimental

Single crystals of $C_{16}H_{38}N_4O_{15}Pd_2$ **[threoninePd]** were **grown by allowing an acetone/water solution to evaporate slowly**. A suitable crystal was selected and mounted on a fiber on a **Xcalibur, Sapphire3, Gemini ultra** diffractometer. The crystal was kept at 100.00(10) K during data collection. Using Olex2 [1], the structure was solved with the SHELXT [2] structure solution program using Intrinsic Phasing and refined with the SHELXL [3] refinement package using Least Squares minimisation.

1. Dolomanov, O.V., Bourhis, L.J., Gildea, R.J, Howard, J.A.K. & Puschmann, H. (2009), J. Appl. Cryst. 42, 339-341.
2. Sheldrick, G.M. (2015). Acta Cryst. A71, 3-8.
3. Sheldrick, G.M. (2015). Acta Cryst. C71, 3-8.

Crystal structure determination of [threoninePd]

Crystal Data for $C_{16}H_{38}N_4O_{15}Pd_2$ (M =739.30 g/mol): orthorhombic, space group $P2_12_12_1$ (no. 19), a = 10.6869(2) Å, b = 11.3234(3) Å, c = 21.0572(3) Å, V = 2548.17(9) Å^3, Z = 4, T = 100.00(10) K, μ(Mo Kα) = 1.490 mm^{-1}, $Dcalc$ = 1.927 g/cm^3, 22934 reflections measured (6.516° ≤ 2Θ ≤ 65.618°), 8597 unique (R_{int} = 0.0555, R_{sigma} = 0.0814) which were used in all calculations. The final R_1 was 0.0404 (I > 2σ(I)) and wR_2 was 0.0691 (all data).

Refinement model description

Number of restraints - 0, number of constraints - unknown.

Details:

```
1. Fixed Uiso
 At 1.2 times of:
  All C(H) groups, All N(H,H) groups
 At 1.5 times of:
  All C(H,H,H) groups, All O(H) groups, All O(H,H) groups
2.a Free rotating group:
 O3W(H3WA,H3WB), O2W(H2WA,H2WB), O1W(H1WA,H1WB)
2.b Ternary CH refined with riding coordinates:
 C22(H22), C32(H32A), C62(H62A), C72(H72), C21(H21), C31(H31A), C61(H61A),
 C71(H71)
2.c Secondary CH2 refined with riding coordinates:
 N12(H12A,H12B), N22(H22A,H22B), N11(H11A,H11B), N21(H21A,H21B)
2.d Idealised Me refined as rotating group:
 C42(H42A,H42B,H42C), C82(H82A,H82B,H82C), C41(H41A,H41B,H41C), C81(H81A,H81B,
 H81C)
2.e Idealised tetrahedral OH refined as rotating group:
 O32(H32), O62(H62), O31(H31), O61(H61)
```

F. Complex (17) cis-bis-(aspartate)palladium(II)

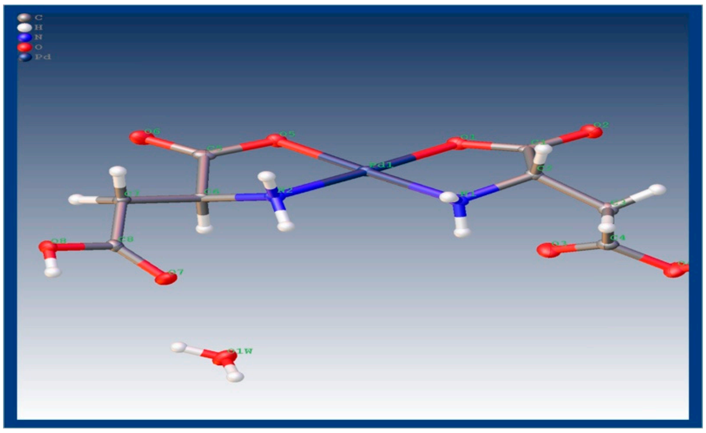

Table S1. Crystal data and structure refinement for dh1-47abs.

Identification code	dh1-47abs
Empirical formula	$C_8H_{14}N_2O_9Pd$
Formula weight	388.61
Temperature/K	100.1
Crystal system	monoclinic
Space group	$P2_1$
a/Å	10.4449(3)
b/Å	5.22088(12)
c/Å	11.6215(3)
α/°	90
β/°	105.340(3)
γ/°	90
Volume/Å^3	611.16(3)
Z	2

ρ_{calc}g/cm^3	2.112
μ/mm^{-1}	1.568
F(000)	388.0
Crystal size/mm^3	0.1677 × 0.0841 × 0.0605
Radiation	Mo Kα (λ = 0.71073)
2Θ range for data collection/°	7.272 to 60.108
Index ranges	-14 ≤ h ≤ 14, -7 ≤ k ≤ 7, -16 ≤ l ≤ 16
Reflections collected	11992
Independent reflections	3571 [R_{int} = 0.0415, R_{sigma} = 0.0443]
Data/restraints/parameters	3571/1/193
Goodness-of-fit on F^2	1.018
Final R indexes [I>=2σ (I)]	R_1 = 0.0233, wR_2 = 0.0432
Final R indexes [all data]	R_1 = 0.0275, wR_2 = 0.0446
Largest diff. peak/hole / e Å^{-3}	0.76/-0.44
Flack parameter	-0.034(19)

Table S2. Fractional Atomic Coordinates (×10^4) and Equivalent Isotropic Displacement Parameters (Å^2×10^3) for dh1-47abs. U_{eq} is defined as 1/3 of of the trace of the orthogonalised U_{IJ} tensor.

Atom	*x*	*y*	*z*	U(eq)
Pd1	10138.8(2)	17274.3(6)	3085.6(2)	8.99(6)
O1	11708(3)	19404(5)	2905(2)	12.1(5)
O2	13595(3)	19092(5)	2360(2)	14.9(6)
O3	11017(3)	17965(5)	75(2)	18.3(7)
O4	12293(3)	16490(5)	-1015(2)	19.5(7)
O5	9484(3)	19886(5)	4072(2)	12.7(5)
O6	7834(3)	20539(5)	4924(2)	17.8(6)
O7	5858(3)	13264(5)	2363(2)	19.5(6)
O8	4946(3)	12640(6)	3879(2)	14.7(7)
N1	10840(3)	14708(6)	2117(3)	10.8(6)
N2	8603(3)	15214(5)	3316(3)	10.1(6)
C1	12531(4)	18149(7)	2470(3)	9.6(7)
C2	12231(4)	15376(7)	2139(3)	9.6(7)
C3	12562(4)	14695(7)	971(3)	11.3(7)
C4	11895(4)	16524(7)	-12(3)	12.6(8)
C5	8342(4)	19309(7)	4270(3)	11.7(7)
C6	7606(3)	17034(10)	3554(3)	10.3(7)
C7	6609(4)	15819(7)	4137(3)	10.7(7)
C8	5785(4)	13798(7)	3367(3)	10.8(7)
O1W	5312(3)	9219(6)	633(3)	20.6(6)

Table S3. Anisotropic Displacement Parameters (Å^2×10^3) for dh1-47abs. The Anisotropic displacement factor exponent takes the form: $-2\pi^2[h^2a^{*2}U_{11}+2hka^*b^*U_{12}+\ldots]$.

Atom	U_{11}	U_{22}	U_{33}	U_{23}	U_{13}	U_{12}
Pd1	9.57(10)	6.51(10)	10.16(10)	0.06(14)	1.34(8)	-1.30(16)
O1	12.7(13)	9.1(12)	13.9(13)	1.6(10)	2.4(11)	-1.8(11)
O2	13.4(14)	14.5(13)	16.9(14)	-1.6(10)	4.1(11)	-5.3(11)
O3	15.9(14)	22.3(17)	15.8(13)	5.9(10)	2.7(12)	5.0(11)
O4	21.3(16)	24.9(17)	13.2(13)	5.0(10)	6.2(12)	5.0(11)
O5	12.2(14)	9.1(13)	16.6(14)	-1.9(10)	3.3(12)	-1.0(10)
O6	16.0(15)	14.3(14)	21.5(15)	-6.8(11)	2.2(12)	2.4(12)
O7	17.6(15)	27.8(15)	13.9(13)	-8.1(11)	5.9(12)	-6.8(11)
O8	11.6(12)	18(2)	15.3(12)	-2.0(12)	5.4(10)	-4.0(12)
N1	11.0(16)	7.7(14)	13.0(15)	0.8(11)	1.7(13)	-2.0(12)
N2	12.1(16)	5.2(14)	12.0(15)	-0.9(11)	1.6(13)	0.6(12)
C1	11.5(19)	9.2(16)	6.0(17)	1.7(12)	-1.4(15)	0.9(13)
C2	8.3(18)	7.9(18)	11.6(19)	1.2(14)	0.8(15)	0.3(14)
C3	11.7(18)	7.7(16)	13.8(18)	2.8(13)	2.2(15)	0.7(14)

Atom	U_{11}	U_{22}	U_{33}	U_{23}	U_{13}	U_{12}
C4	13.2(19)	11.8(18)	11.6(17)	1.4(12)	1.1(15)	-1.3(13)
C5	12.2(18)	8.5(17)	11.7(18)	1.4(13)	-1.6(15)	1.8(14)
C6	9.9(14)	9(2)	11.6(14)	-2.9(17)	1.9(11)	-5.8(19)
C7	10.7(18)	11.4(17)	9.4(17)	-2.4(13)	1.5(14)	-0.3(14)
C8	8.3(17)	9.0(16)	15.0(18)	0.2(13)	2.8(14)	0.7(14)
O1W	25.0(17)	21.6(15)	17.3(15)	-7.1(12)	9.4(13)	-5.4(13)

Table S4. Bond Lengths for dh1-47abs.

Atom	Atom	Length/Å	Atom	Atom	Length/Å
Pd1	O1	2.037(3)	O7	C8	1.222(4)
Pd1	O5	2.014(3)	O8	C8	1.328(4)
Pd1	N1	2.009(3)	N1	C2	1.488(5)
Pd1	N2	2.008(3)	N2	C6	1.488(5)
O1	C1	1.286(5)	C1	C2	1.510(6)
O2	C1	1.253(5)	C2	C3	1.529(5)
O3	C4	1.211(4)	C3	C4	1.510(5)
O4	C4	1.337(4)	C5	C6	1.535(6)
O5	C5	1.309(4)	C6	C7	1.523(5)
O6	C5	1.219(4)	C7	C8	1.499(5)

Table S5. Bond Angles for dh1-47abs.

Atom	Atom	Atom	Angle/°	Atom	Atom	Atom	Angle/°
O5	Pd1	O1	95.30(11)	C1	C2	C3	111.7(3)
N1	Pd1	O1	83.33(11)	C4	C3	C2	111.0(3)
N1	Pd1	O5	178.49(13)	O3	C4	O4	119.9(3)
N2	Pd1	O1	178.19(12)	O3	C4	C3	122.5(3)
N2	Pd1	O5	83.28(11)	O4	C4	C3	117.6(3)
N2	Pd1	N1	98.07(12)	O5	C5	C6	115.6(3)
C1	O1	Pd1	113.7(2)	O6	C5	O5	123.8(3)
C5	O5	Pd1	114.0(2)	O6	C5	C6	120.5(3)
C2	N1	Pd1	109.5(2)	N2	C6	C5	108.7(3)
C6	N2	Pd1	107.8(2)	N2	C6	C7	114.3(4)
O1	C1	C2	118.3(4)	C7	C6	C5	112.6(3)
O2	C1	O1	122.9(3)	C8	C7	C6	112.3(3)
O2	C1	C2	118.7(4)	O7	C8	O8	122.6(3)
N1	C2	C1	111.4(3)	O7	C8	C7	124.1(3)
N1	C2	C3	112.3(3)	O8	C8	C7	113.3(3)

Table S6. Hydrogen Bonds for dh1-47abs.

D	H	A	d(D-H)/Å	d(H-A)/Å	d(D-A)/Å	D-H-A/°
O4	H4	O1W^{1}	0.77(5)	1.96(5)	2.697(4)	161(5)
O8	H8	O2^{2}	0.79(4)	1.90(4)	2.687(4)	173(5)
N1	H1A	O1^{3}	0.89	2.17	2.982(4)	150.4
N1	H1B	O3^{4}	0.89	2.09	2.909(4)	151.8
N2	H2B	O5^{3}	0.89	2.31	2.988(4)	133.4
O1W	H1WA	O1W^{5}	0.88(5)	2.10(5)	2.982(3)	175(4)
O1W	H1WB	O2^{2}	0.82(5)	2.22(5)	3.025(4)	169(5)

^{1}2-X,1/2+Y,-Z; ^{2}-1+X,-1+Y,+Z; ^{3}+X,-1+Y,+Z; ^{4}2-X,-1/2+Y,-Z; ^{5}1-X,-1/2+Y,-Z.

Table S7. Torsion Angles for dh1-47abs.

A	B	C	D	Angle/°	A	B	C	D	Angle/°
Pd1	O1	C1	O2	-175.7(3)	O5	C5	C6	N2	30.2(4)
Pd1	O1	C1	C2	1.0(4)	O5	C5	C6	C7	157.8(3)
Pd1	O5	C5	O6	174.0(3)	O6	C5	C6	N2	-153.6(3)
Pd1	O5	C5	C6	-9.9(4)	O6	C5	C6	C7	-26.0(5)
Pd1	N1	C2	C1	-20.5(3)	N1	C2	C3	C4	73.0(4)

A	B	C	D	Angle/°	A	B	C	D	Angle/°
Pd1	N1	C2	C3	-146.6(2)	N2	C6	C7	C8	-61.9(4)
Pd1	N2	C6	C5	-34.5(3)	C1	C2	C3	C4	-53.0(4)
Pd1	N2	C6	C7	-161.2(2)	C2	C3	C4	O3	-13.8(5)
O1	C1	C2	N1	13.3(5)	C2	C3	C4	O4	167.2(3)
O1	C1	C2	C3	139.7(3)	C5	C6	C7	C8	173.5(3)
O2	C1	C2	N1	-169.8(3)	C6	C7	C8	O7	-3.3(5)
O2	C1	C2	C3	-43.4(5)	C6	C7	C8	O8	177.4(3)

Table S8 Hydrogen Atom Coordinates (Å×10[4]) and Isotropic Displacement Parameters (Å[2]×10[3]) for dh1-47abs.

Atom	*x*	*y*	*z*	U(eq)
H4	12880(50)	15600(90)	-1010(40)	29
H8	4600(50)	11600(90)	3400(40)	22
H1A	10814.08	13146.07	2416.65	13
H1B	10336.46	14710.58	1367.84	13
H2A	8245.04	14298.27	2664.79	12
H2B	8875.68	14144.84	3928.64	12
H2	12812.51	14340.4	2768.38	11
H3A	13515.62	14763.14	1087.21	14
H3B	12269.25	12961.13	741.22	14
H6	7100.62	17705.82	2779.51	12
H7A	7081.8	15062.41	4892.49	13
H7B	6028.54	17140.1	4301.18	13
H1WA	5150(40)	7690(100)	300(40)	31
H1WB	4920(50)	9340(100)	1150(40)	31

Experimental

Single crystals of $C_8H_{14}N_2O_9Pd$ **[dh1-47abs]** were **grown by allowing an acetone/water solution to evaporate slowly**. A suitable crystal was selected and and mounted on a fiber on a **Xcalibur, Sapphire3, Gemini ultra** diffractometer. The crystal was kept at 100.1 K during data collection. Using Olex2 [1], the structure was solved with the SHELXT [2] structure solution program using Intrinsic Phasing and refined with the SHELXL [3] refinement package using Least Squares minimisation.

1. Dolomanov, O.V., Bourhis, L.J., Gildea, R.J, Howard, J.A.K. & Puschmann, H. (2009), J. Appl. Cryst. 42, 339-341.
2. Sheldrick, G.M. (2015). Acta Cryst. A71, 3-8.
3. Sheldrick, G.M. (2015). Acta Cryst. C71, 3-8.

Crystal structure determination of [dh1-47abs]

Crystal Data for $C_8H_{14}N_2O_9Pd$ (M =388.61 g/mol): monoclinic, space group P2$_1$ (no. 4), a = 10.4449(3) Å, b = 5.22088(12) Å, c = 11.6215(3) Å, β = 105.340(3)°, V = 611.16(3) Å^3, Z = 2, T = 100.1 K, μ(Mo Kα) = 1.568 mm^{-1}, $Dcalc$ = 2.112 g/cm^3, 11992 reflections measured (7.272° ≤ 2Θ ≤ 60.108°), 3571 unique (R_{int} = 0.0415, R_{sigma} = 0.0443) which were used in all calculations. The final R_1 was 0.0233 (I > 2σ(I)) and wR_2 was 0.0446 (all data).

Refinement model description

Number of restraints - 1, number of constraints - unknown.

Details:

```
1. Fixed Uiso
 At 1.2 times of:
  All C(H) groups, All C(H,H) groups, All N(H,H) groups
 At 1.5 times of:
  All O(H) groups, All O(H,H) groups
2.a Ternary CH refined with riding coordinates:
 C2(H2), C6(H6)
2.b Secondary CH2 refined with riding coordinates:
 N1(H1A,H1B), N2(H2A,H2B), C3(H3A,H3B), C7(H7A,H7B)
```

These reports have been created with Olex2, compiled on 2020.11.12 svn.r5f609507 for OlexSys. Please let us know if there are any errors or if you would like to have additional features.
